# Insight into Classification and Risk Stratification of Head and Neck Squamous Cell Carcinoma in Era of Emerging Biomarkers with Focus on Histopathologic Parameters

**DOI:** 10.3390/cancers14225514

**Published:** 2022-11-10

**Authors:** Antti A. Mäkitie, Abbas Agaimy, Alhadi Almangush

**Affiliations:** 1 Department of Otorhinolaryngology—Head and Neck Surgery, University of Helsinki and Helsinki University Hospital, 00029 Helsinki, Finland; 2 Division of Ear, Nose and Throat Diseases, Department of Clinical Sciences, Intervention and Technology, Karolinska Institutet and Karolinska Hospital, 17176 Stockholm, Sweden; 3 Research Program in Systems Oncology, Faculty of Medicine, University of Helsinki, 00014 Helsinki, Finland; 4 Institute of Pathology, Friedrich-Alexander University Erlangen-Nürnberg (FAU), Comprehensive Cancer Center (CCC) Erlangen-EMN, University Hospital Erlangen, 91054 Erlangen, Germany; 5 Department of Pathology, University of Helsinki, 00014 Helsinki, Finland; 6 Department of Oral and Maxillofacial Diseases, University of Helsinki, 00014 Helsinki, Finland; 7 Department of Pathology, University of Turku, 20521 Turku, Finland; 8 Faculty of Dentistry, Misurata University, Misurata 2478, Libya

**Keywords:** biomarker, head and neck squamous cell carcinoma (HNSCC), tumor–node–metastasis (TNM) staging system

## Abstract

**Simple Summary:**

Patients with head and neck squamous cell carcinoma (HNSCC) might present with different clinical behaviors, even when classified at the same stage. This perspective highlights the recent findings on prognostic biomarkers that can aid in improving the staging of HNSCC. This was conducted with an aim of subsequently improving prognostic stratification and, hence, treatment planning.

**Abstract:**

Tumor-node-metastasis (TNM) staging system is the cornerstone for treatment planning of head and neck squamous cell carcinoma (HNSCC). Many prognostic biomarkers have been introduced as modifiers to further improve the TNM classification of HNSCC. Here, we provide an overview on the use of the recent prognostic biomarkers, with a focus on histopathologic parameters, in improving the risk stratification of HNSCC and their application in the next generation of HNSCC staging systems.

## 1. Introduction

Head and neck squamous cell carcinoma (HNSCC) is a common and heterogenous malignancy in regard to etiology, pattern of progression and survival [1]. Risk factors of HNSCC include tobacco and/or alcohol consumption. Additionally, human papillomavirus (HPV) infection is an important risk factor, specifically for the oropharyngeal subgroup The incidence of HNSCC has increased in many countries according to recent reports [2,3]. Furthermore, a large number of HNSCC cases are usually diagnosed at an advanced stage and will thus have poor survival [1]. Therefore, the mortality rate of some head and neck cancer subsites still remains high [3]. Various non- or mini-invasive methods are currently under investigation to aid in early diagnosis [4]. For example, blood or exhaled breath sampling and analysis may prove as promising potential novel tools during cancer screening [4,5]. In moving towards establishing a complete understanding of HNSCC, it is necessary to consider a multidisciplinary process traditionally involving a collaboration of head and neck surgeons, radiologists, and pathologists. This will aid in the proper management of HNSCC. Thus, clinical decision making should be based on the conclusion of clinical, radiological, and pathological evaluations of the tumor and the patient status.

The next step after diagnosis of HNSCC is to design treatment planning. Notably, cases sharing the same tumor–node–metastasis (TNM) stage usually receive the same treatment, although they may present with dramatically different clinical behavior [6]. Of note, recent systematic reviews and meta-analyses have highlighted many promising biomarkers that have the potential to prove useful in HNSCC prognostication. However, none of them are included in daily practice due to differences among the evaluation methods and staining protocols, or otherwise a lack of validation [7,8,9,10]. There were major changes in the techniques used to stage HNSCC in the latest release of the American Joint Committee on Cancer (i.e., 8th edition of the AJCC-TNM classification) manual [11,12]. Furthermore, many recent studies have proposed further refinement of the HNSCC classification based on the clinical significance of the recently emerged prognostic biomarkers.

## 2. Emerging Prognostic Biomarkers of HNSCC

The field of biomarker cancer research has introduced numerous candidates (Table 1) that have been proven to be associated with the prognosis of HNSCC using different techniques [13]. Among these, immunohistochemistry is an essential technique in the diagnostics and prognostication of HNSCC due to its facilitation of qualitative information. For example, immunohistochemical expression of programmed cell death ligand-1 (PD-L1) has shown a significant value in predicting response to treatment [14,15], with recent studies comparing the methods of assessment including Tumor Proportion Score (TPS) and Combined Positive Score (CPS) for scoring of PD-L1 [16]. Other techniques, such as genomic approaches including microarrays and quantitative PCR, provide technical usefulness. However, their role in the prognostication has remained limited due to the lack of validation studies from large homogenous cohorts into tumor location and tumor stage. Of note, circulating tumor DNA (ctDNA) has been analyzed in HNSCC, showing a clinical significance in diagnosis and risk assessment of HNSCC. These findings require further validation [17,18]. In addition, long non-coding RNAs (lncRNA) have been studied in HNSCC, with accumulating evidence suggesting they have a powerful prognostic value [19].

During the last decade, and with the use of the above-mentioned methods/techniques, several molecules have been studied for their significance in initiation and/or progression of HNSCC [13]. However, few molecular biomarkers are assessed in tumor tissue specimens during routine pathology reporting on some subsites of HNSCC. In oropharyngeal subsite, as an example, p16 (INK4A) immunohistochemistry is a widely used and generally accepted surrogate tool to identify HPV association and therefore, to classify oropharyngeal SCC as either associated with the viral infection (i.e. HPV positive that usually has better prognosis) or not associated (i.e. HPV negative having poor survival) [33]. Although using polymerase chain reactions or in situ hybridization can result in a more accurate result in such a HPV status-based classification [36], it has now been widely accepted that p16 immunohistochemistry, if interpreted in the appropriate anatomic (orppahyngeal location and moprhological (HPV-typical or compatile mrophology) context using standardized assessment (usually block-type expression in >80% tumor cells), correlates highly with the presence of oncogenically active HPV infection. Indeed, p16 status is the basis of the current TNM classification system.

Focusing on body fluids, namely blood and saliva, recent research on HNSCC has highlighted the prognostic significance of many molecular-based biomarkers [37,38,39]. The assessment of such parameters has the advantages of being non-invasive and easily assessed preoperatively. Blood biomarkers, including neutrophil-to-lymphocyte ratio (NLR) and platelet-to-lymphocyte ratio (PLR), have been widely studied in HNSCC. Evidence from recent meta-analysis indicated that NLR is an important prognostic factor in HNSCC, while PLR was not of similar significance in survival prediction [40,41]. Similarly, salivary biomarkers have been studied in HNSCC, with a greater focus on oral SCC [37]. However, molecular biomarkers from body fluids are not yet considered in daily practice. The challenges in biomarker development, such as false negativity, false positivity and small size of cohorts, were widely noticed in studies of HNSCC [13]. In addition, the cost of the molecular biomarkers is a challenge. Therefore, the main focus in daily practice remains on histopathologic-based prognostic markers.

## 3. Histologic Markers Proposed Recently to Improve Risk Stratification of HNSCC

Conventional tumor histomorphology and anatomic characteristics of the tumor are the cornerstones of risk stratification methods in the analysis of many tumors, including HNSCC [23]. Daily practice in pathology reporting considers depth of invasion, perineural invasion, tumour grade, status of surgical margins, lymphovascular invasion, and local extension to adjacent structures (e.g., muscular and bone invasion). Some of these parameters have a clinical relevance in all subsites of HNSCC, e.g., perineural invasion [24] and lymphovascular invasion [25,26]; while some parameters are clinically more important for specific subsites, such as depth of invasion in oral SCC [27,28].

**Figure 1:**
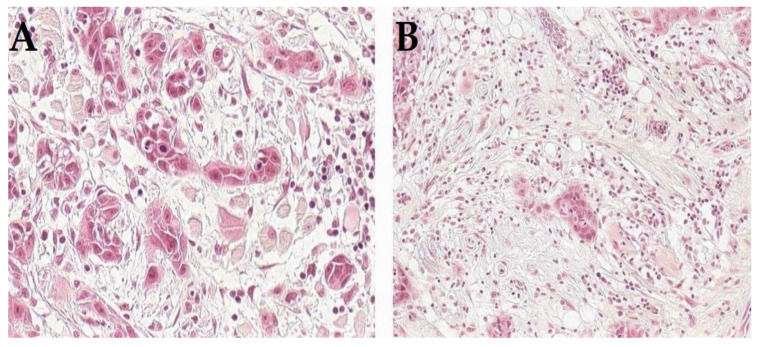
Histologic appearance of HNSCC with adverse prognostic features such as tumor budding (**A**) or high amount of stroma (**B**).

Furthermore, recent research has underlined many markers that can be assessed using HE-stained sections. Here, we will underline those that have been reported as modifiers to further develop and refine the classification of HNSCC, both for TNM staging and histologic grading. These include tumor-infiltrating lymphocytes (TILs), tumor–stroma ratio and tumor budding (Figure 1) since they have been widely studied in HNSCC [10,29,30] and proposed for incorporation into the staging and/or grading systems. For example, the TNM-Immune system has been recently introduced as a superior prognostic classifier compared with the traditional TNM staging system [42]. The fact that immune cells are key components in the tumor microenvironment has led to large research efforts to identify immune biomarkers. Specifically, TILs have been among the widely studied immune cells, and their prognostic significance has been repeatedly reported in HNSCC [29,43,44,45]. Interestingly, our recent study proposed a modification for the TNM staging by incorporating the immune response (as assessed by the score of TILs in HE-stained sections), i.e., “TNM-Immune system”, and which showed a promising value in improving risk stratification of early oral tongue SCC [46]. In addition, a recent study by Bjerkli and colleagues [31] combined lymphocytic infiltrate and tumor differentiation in a “histo-score” that showed a prognostic value superior to the traditional tumor differentiation grade in assessments of oral tongue SCC. Similarly, the combination of infiltrating lymphocytes with type of histology has shown a promising prognostic value in a recent study of nasopharyngeal cancer [44].

The significance of stromal microenvironment has been recently underlined in HNSCC [10,47]. Thus, assessing prognostic markers related to the tumor–stroma can provide prognostic information of important clinical relevance. Of note, the tumor–stroma ratio has been recently reported to provide reliable prognostic value in different subsites of HNSCC, according to our recent systematic review [10]. In tongue SCC, a recent study by Mascitti et al. [32] proposed adding tumor–stroma ratio to the 8th edition of the AJCC-TNM classification to improve survival prediction. Remarkably, a Tumor–Stroma Node Metastasis (TSNM) (TSNM) staging system has also been recently proposed for gastric cancer [48], breast cancer [49] and esophageal cancer [50] and has demonstrated better ability in risk stratification compared with the traditional TNM staging system. Indeed, this proposal still needs to be further studied, as the addition of tumor–stroma ratio to TNM classification of HNSCC is still a preliminary proposal from a single study.

Tumor budding has been defined as single cancer cell/s or small cluster/s of less than five cancer cells, and it has become increasingly reported as a promising prognostic marker in solid tumors [51]. Tumor budding represents tumor cell dissociation at the invasion front and has shown an association with epithelial-to-mesenchymal transition [52,53,54]. It is well-documented that tumor budding has significant clinical relevance in HNSCC [30] and other solid cancers as well, including in particular colorectal cancer where it has been very extensively studied [51]. Of note, the significance of tumor budding in HNSCC has been confirmed in a recent meta-analysis [55]. Many recent studies have incorporated tumor budding as a part of the grading system in studies of oral tongue SCC [56] oral SCC [57], laryngeal SCC and hypopharyngeal SCC [58].

It is important to take into consideration that tumor heterogeneity might represent a challenge in regard to representativity of the tumor sample when assessing these emerging prognostic biomarkers in preoperative biopsies. This is a common limitation for most of histopathological markers. However, recent studies have shown a good concordance in small biopsies and resection samples for some of these histopathologic parameters (e.g., TILs in oropharyngeal SCC [59] and tumor budding in oral SCC [60]) in cases with good-quality representative biopsies. This issue, however, still needs more comparative studies to be conducted in large cohorts.

Regarding histopathological grading, the current WHO classification has adopted a dichotomous approach to oropharyngeal SCC, with conventional (tobacco-associated, HPV-negative) tumors to be graded as usual while HPV-positive (p16-positive) tumors should not be graded. Inclusion of mixed etiology tumors in historical series with HPV-positive tumors (usually assigned Grade 3, but showing better outcome) and lower-grade (G1/G2) conventional tumors that are by definition virtually HPV-negative, is likely responsible for a great amount of the contradictory grading-related survival data. The proposal of two different sets of TNM categories for p16-positive (HPV-related) and p16-negative (HPV-negative) oropharyngeal SCC represents a first step in adopting an individualized staging risk stratification system for HNSCC.

## 4. Discussion

Cancer management in general is a medical field with many multidisciplinary aspects. The current comprehensive evaluation of HNSCC patients for decision making regarding management includes several patient-related factors. These include, but are not limited to, demographics [20], performance status [61], nutritional status [21], and comorbidities [22]. Diagnosis needs to be regarded as a summary of many factors and not just the ‘histo-patho-molecular’ diagnosis. The aforementioned factors will provide more comprehensive and individualized diagnostic and prognostic information. Clinical decision making in head and neck oncology aims to offer the best options for personalized management of the patient. Up-to-date TNM staging remains a major determinant for prognosis of HNSCC and therefore, it is widely considered during treatment planning. Aiming towards a more personalized cancer staging, the 8th edition of the AJCC-TNM classification staging manual introduced major changes in the staging system of some anatomical subsites of HNSCC. The most significant modifications from the previous 7th edition were the separate staging algorithm for high-risk human papillomavirus (HPV)-associated cancer of the oropharynx; the addition of extra-nodal extension to the lymph node category (N) in all but the viral-related cancers; and the inclusion of depth of invasion in the evaluation of the tumor category (T) of oral cancers. During preoperative staging and treatment planning, however, it can be sometimes challenging to exactly evaluate these modifiers that are included in the 8th edition of the AJCC-TNM classification. Therefore, it is of research interest and clinical importance to take into account the strategies that can aid in the best preoperative assessment of these modifiers. Similarly, it is important to identify the optimal cutoff for the number of metastatic lymph nodes. This can be considered to refine the N-staging, as has been reported in recent studies that proposed new classifications mostly redefining the optimal cutoff in the number of metastatic nodes in HPV-negative HNSCC [62,63]. Such refinement can aid in overcoming the limitations of the current N-staging. Further, it is necessary to consider the prognostic significance of nodal yield that has also been highlighted in studies of HNSCC [64].

In the daily practice of pathology, histologic risk stratification/classification of HNSCC is routinely determined using HE staining and mainly considers the degree of differentiation. HNSCC can be categorized for the majority of subsites into two groups: keratinizing SCC (usually showing squamous differentiation) and non-keratinizing SCC (usually showing limited maturation) [65]. Conventional HNSCCs are further histopathologically classified as well-, moderately and poorly differentiated tumors. Although this classification is classically reported by pathology reports, it has a limited value in the treatment planning of certain HNSCCs. For instance, in oral SCC, the aforementioned three groups do not associate well with the clinical outcome [65]. Therefore, recent studies have attempted to revise this system by, for example, incorporating tumor budding [56,58,66] or lymphocytic infiltrate [31,44] as part of histologic classification of HNSCC. Such recent modifications have showed superior prognostic value compared with the conventional WHO grading scheme and therefore remain necessary for consideration in further validation studies. In addition to histologic conventional histologic grading, there are other histopathologic parameters (e.g., pattern of invasive front, perineural invasion and lymphovascular invasion) that are widely evaluated in HNSCC using HE staining and can aid in understanding the behavior of each individual HNSCC case.

While the 8th edition of AJCC-TNM classification has incorporated the above-mentioned modifiers, there are other emerging prognostic biomarkers that have shown clinical significance in HNSCC and can be routinely evaluated and considered during the classification in daily diagnostics. Among these, immune-related biomarkers have been underlined in recent research and can be utilized for another kind of an important stratification in many cancers including HNSCC as either immune-hot (highly infiltrated) or immune-cold (non-infiltrated) tumors based on quantification of specific molecules such as CD3 and CD8 [34,67]. Of note, such findings on the prognostic significance of immune biomarkers have been approved in recent meta-analyses [35,68,69,70]. Further, accumulating evidence from recent studies indicates that HNSCC immune-hot tumors with a high infiltration of immune cells have an improved survival rate compared with immune-cold tumors, as simply assessed on HE-stained sections [29]. Furthermore, “hot” solid tumors with prominent CD8 infiltrate were reported to have a good response to immunotherapy [71]. Better understanding of immune microenvironment and immune classifier/s of HNSCC is of a high clinical significance, as it can identify suitable patients to benefit from immunotherapy [72]. Immune score has been introduced as a novel classification in colorectal cancer [73], and emerging evidence is indicating that such a score is of a significant value in HNSCC [70]. However, the accumulated evidence about novel IHC-based biomarkers in HNSCC still requires further validation to be included in daily practice.

There are a number of “Major Challenges” that need to be taken into account when considering the biomarkers of HNSCC. This includes, for example, the heterogenity of HNSCC tumors in regard to tumor location (subsites such as oral cavity, oropharynx, larynx, etc.) and risk factors (smoking/alcohol use vs. virus-related). Such heterogenity influences survival prediction and risk stratification, as one biomarker can be reliable for one subsite but not for the other subsite/s. Indeed, there are somehow universal prognostic markers that have been successfully predictive in more than one subsite [29,30]. Therefore, conducting a sub-analysis of each subsite separately is mandatory. The second challenge is defining the cutoff points in regard to dividing the cases into risk groups. For example, using a cutoff point of 20% has been widely used when considering TILs for risk stratification for HNSCC, but a cutoff point of 5% has been specifically suggested for nasopharyngeal cancer [29]. Thus, reporting the results of different cutoff points in each study can aid in recognizing the most suitable threshold for risk stratification. The third challenge is the small number of included cases, specifically in single-institution studies. Such small cohorts might be one of the reasons for conflicting findings on the studied biomarker/s and have caused difficulty in reaching a definitive conclusion on the significance of the prognostic biomarkers [7]. Overcoming these challenges in future studies is warranted to clarify if the introduced markers are suitable for all subsites of the head and neck region and to find the optimal cutoff point for every marker. Large multicenter cohorts are preferred for such studies.

## 5. Conclusions

From the anatomical, morphological and biological aspects pointed out above, it can be concluded that many histological, immunohistochemical, immunological, and molecular markers have been proposed for incorporation into the staging and grading systems of various HNSCC subsites. They might thus be further eligible to be considered in the next generation of the clinical and histological classifications. The improvement in the performance of 8th edition of the AJCC-TNM classification, together with the potentially forthcoming markers that can be implemented in routine pathology, is an important step towards a more personalized management of HNSCC. Namely, tumor budding, TSR and TILs have been simple to assessed and have high clinical relevance, and thus are important to considered in HNSCC pathology reporting. Taking into consideration such cancer-related and stromal-related prognostic biomarkers, at the same time, can aid in more accurate prediction. The current TNM staging system can be further modified/revised according to recent reports as discussed above. Future research is warranted to validate the proposed modifications in large multi-institutional studies.

## Figures and Tables

**Table 1:** Summary of prognostic classifiers/markers in head and neck squamous cell carcinoma.

Category	Example of Prognostic Parameter/Marker	References
**Demographic factors**	Age, gender, race, socioeconomic status, etc.	[20]
**Nutritional factors**	Pretreatment nutritional index	[21]
**Comorbidity**	Osaka head and neck comorbidity index	[22]
**TNM classification**	Tumor size, lymph node status, and distant metastasis	[11,12]
**Histology-based biomarkers**	Tumor grade, perineural invasion, tumor budding, tumor–stroma ratio, tumor-infiltrating lymphocytes, etc.	[23,24,25,26,27,28,29,30,31,32]
**Immunohistochemical-based biomarkers**	p16, CD3, CD8, etc.	[33,34,35]
**Liquid biopsy analysis**	Circulating tumor cells	[17,18]
